# Lessons learned from the COVID-19 pandemic in Latin America: A Data Science standpoint

**DOI:** 10.1371/journal.pone.0324171

**Published:** 2025-05-30

**Authors:** Jéssica Villar, Paula Maçaira, Fernanda A. Baião

**Affiliations:** Department of Industrial Engineering, Pontifical Catholic University of Rio de Janeiro, Rio de Janeiro, RJ, Brazil; Norbert Wiener University, PERU

## Abstract

In the 21st century, the world has faced the devastating impacts of three acute respiratory diseases: Middle East respiratory Syndrome (MERS), Severe Acute respiratory Syndrome (SARS), and COVID-19, which evolved into a pandemic. These diseases have not only caused a large number of deaths but have also damaged the economies of the affected regions. Particularly, countries in the Latin American and Caribbean (LAC) region have faced additional challenges due to more significant social inequalities, limited access to Healthcare services, and precarious living conditions, and it is not clear how these challenges impacted the effects of the mitigation actions adopted by each country. However, this understanding is crucial to guide measures to mitigate Health and socioeconomic impacts if (or when) new acute respiratory diseases emerge, especially in these countries. A retrospective study was conducted to model the dynamics of variation in COVID-19 mortality in LAC countries and to analyze its association with vaccination strategies, containment measures, mobility restrictions, and socioeconomic factors. The study methodology applied clustering techniques that revealed two distinct clusters based on sociodemographic characteristics, followed by the application of XGBoost to model the dynamics of variation in deaths in the countries of each cluster over time. Finally, the SHAP Values technique was applied to understand the associations between mortality and factors such as vaccination, containment measures, and mobility restrictions. The study provides evidence that economic support and the completion of the vaccination scheme were especially relevant in reducing COVID-19 mortality. Two distinct groups of countries were detected, one characterized by a greater vulnerability. The most important interventions for understanding COVID-19 mortality varied between pre- and post-vaccination periods. In the pre-vaccination period, containment measures were the most important interventions for mortality in the less vulnerable countries, while population mobility was more important in the more vulnerable countries. In the post-vaccination period, vaccination coverage was the most important intervention for mortality in the less vulnerable countries, while containment measures impacted the more vulnerable countries.

## Introduction

Since 2001, three new coronaviruses have been responsible for causing respiratory diseases whose spread led to four large-scale outbreaks [1]: the Severe Acute respiratory Syndrome (SARS) in 2003, two Middle East respiratory Syndrome (MERS) - the first in the Middle East in 2012 and the second in South Korea in 2015 -, and the COVID-19 pandemic that began in 2019. As the most recent outbreak, COVID-19 is the current case study for the ongoing challenge of emerging infectious pathogens [2]. Its causing virus, SARS-Cov-2, was first detected in Wuhan, China, in December 2019, and in February 2020, it had already spread to Latin America and the Caribbean (LAC) [3]. By March 2020, every country in LAC had reported infections [4], and by June 2020, LAC accounted for 27% of deaths from COVID-19 worldwide, becoming the region with the highest number of deaths in the world [5].

LAC is the world’s most inequality-ridden region, with 53% of its working population earning their income from informal work [4]. Underfunded state-run hospitals are the only source of medical care for those with informal jobs or unemployed [6]. LAC countries suffer from social inequalities, several localities lacking access to Healthcare services, and poor health outcomes. Overcrowding, limited sanitation, food insecurity, and poor nutrition are common health problems [6]. Urban slums in large LAC cities, such as Bogota, Buenos Aires, Lima, Mexico City, Rio de Janeiro, and São Paulo, are more susceptible to COVID-19 and other infectious diseases due to their high population density [4]. According to [7] and [8], the impacts differ for each country because of that heterogeneity. For example, the effect of COVID-19 will be more devastating in Venezuela than in more developed economies, such as Brazil, due to the Venezuelan humanitarian crisis, spreading many other diseases over the region [7].

Governments implemented several social distancing measures to cope with the COVID-19 pandemic [9]. Those policies were crucial to reduce the spread of the disease, especially before vaccines became available; however, they may also worsen the situation for the poor population, who lack food supplies, cannot work remotely, and rely on manual labour [9]. Lockdowns in Chile positively reduced the number of cases within high-income geographical areas, with no effects in lower-income areas [10]. A possible explanation is that poor people are less likely to comply with social distance measures, as they must work [9]. Thus, mobility restrictions not accompanied by social transfer programs are less likely to be followed by the poorest population [9, 11]. In 2020, despite the demand from WHO that high equitable immunization coverage be prioritized at all levels (national, municipal, and district) [12], the inequality in routine immunization in several LAC countries worsened with the scenario imposed by COVID-19, mainly due to the disproportionate impact on vulnerable populations [12]. A Brazilian study about inequity in COVID-19 vaccination showed that municipalities with low Human Development Index (HDI) had a lower first dose coverage than those with medium and high HDI [13].

Given these disparities and the complex interplay between socioeconomic factors and Public Health measures, it is important to deepen the understanding of these interventions’ specific impacts and effectiveness. Therefore, the objectives of this research are:

1. To understand which and how LAC countries are similar according to their socioeconomic and demographic characteristics;
2. To identify the best lag of days between interventions (vaccination coverage, containment measures, economic support, and population mobility) and COVID-19 mortality;
3. To identify which interventions are most important to understand COVID-19 mortality and how they are associated with COVID-19 mortality;
4. To provide insights into the impacts of vaccination coverage, containment measures, economic support, and population mobility in COVID-19 mortality, providing insights toward the definition of Public Health guidelines in future respiratory diseases.

The research started by clustering countries with similar socioeconomic and demographic characteristics to achieve the proposed objectives. Next, the researchers separated into pre- and post-vaccination periods to model the relationship between variables and COVID-19 deaths in each cluster using XGBoost models. Finally, the association between each independent variable and mortality was interpreted by calculating its SHAP Values. Finally, the results were presented to experts in the Healthcare domain and evaluated concerning their degree of surprise, their potential contribution toward the definition of new Public Health guidelines, and the insights and bottlenecks they identified in the research.

## Materials and methods

### Study design and data sources

This study reports a retrospective analysis of COVID-19 mortality data in LAC countries. We considered its evolution over time and examined how vaccination coverage, containment measures, economic support, and population mobility contributed to the evolution of deaths from COVID-19 in LAC countries, accounting for their socioeconomic differences.

COVID-19 data records were provided by Our World in Data (https://ourworldindata.org/), containing daily data on cases, deaths, and vaccination rates. Data on containment measures was made available by the Oxford Covid-19 Government Response Tracker project (https://www.bsg.ox.ac.uk/research/covid-19-government-response-tracker). Mobility data was obtained from Google Mobility Data (www.google.com/covid19/mobility). Socio-demographic data was collected from Our World in Data, Human Development Reports (https://hdr.undp.org/data-center), and The World Bank (https://data.worldbank.org/indicator/).

The study included data from January 1, 2020, to October 15, 2022. It was analyzed data from all 20 LAC countries (Argentina, Bolivia, Brazil, Chile, Colombia, Costa Rica, Cuba, Ecuador, El Salvador, Guatemala, Haiti, Honduras, Mexico, Nicaragua, Panama, Paraguay, Peru, Dominican Republic, Uruguay, and Venezuela).

### Outcome

The outcome is the COVID-19 mortality rate, calculated as the 7-day daily moving average of COVID-19 deaths per 100,000 population.

### Statistical analysis

All analysis and modelling were performed in Python. Except for the mobility variables, all variables were standardized by the Z-Score method and then by the Min-Max standardization to keep the variable values between 0 and 1. The mobility variables were only standardized by the Z-Score method since these variables can assume negative values.

According to [14], people living in a country face multiple difficulties simultaneously. Therefore, when characterizing a country, it is important not to consider a single dimension (for example, high-income or low-income). Thus, the proposed method addresses various socioeconomic and demographic characteristics of countries through the clustering technique to reduce the dimensionality of the data and create an additional feature for classification, to be used as an adjustment covariate in the modelling stage. Before clustering, an analysis was conducted to detect multicollinearity in the candidate variables for clustering using VIF (Variance Inflation Factor), where variables with a VIF greater than ten were removed [15]. The K-Means [16] and DBSCAN [17] clustering methods were tested, and the silhouette index, the elbow method, and the Calinski-Harabasz score were tested to choose the number of clusters.

To assess the effectiveness of interventions during the pandemic, such as the closure of schools and the percentage of the population that is fully vaccinated, it is important to lag deaths from interventions, as this lag represents the time between the implementation of the measures and the observation of the results of these measures on the number of deaths. This day’s lag was calculated to find the ideal lag between the mortality rate and the interventions. Lags of 21 to 49 days were tested for each cluster, according to [18], whose work applied these lags to assess the effects of non-pharmaceutical interventions (NPIs) on COVID-19 cases and deaths and analyzed the aggregated reality of 32 European countries and found associations after 21 to 49 days. Poisson regressions [19] were applied to each cluster, and each lag was tested to define the best lag. The best lag was chosen based on the AIC. After the lag, a multicollinearity analysis was carried out with the VIF in the intervention variables, and, again, variables with a VIF greater than ten were removed [15].

Poisson regression is a statistical technique used to model count data, where the dependent variable is a count of events that occur in a fixed time or space interval. Based on the Poisson distribution, this regression assumes that the mean of the count is equal to its variance, which makes it particularly suitable for count data that follow this property [19].

An XGBoost (Extreme Gradient Boosting) model [20] was then applied, with the lagged COVID-19 mortality rate as its response variable and a 7-day moving average adjusted by the predominance of each COVID-19 variant per country. The intention was to remove the confounding effects of variants in analyzing the measures that most influenced mortality. The present work extensively experimented with varying parameters to improve the proposed model’s performance.

Finally, the SHAP Values technique [21] was applied to the results of the XGBoost modelling to understand how each feature of the model is associated with mortality from COVID-19.

In the research, experts evaluated the relevance and applicability of the results, aiming to ensure that the conclusions obtained were theoretically robust but also applicable and helpful in a practical context. This validation with experts is crucial, as it allows the results to be interpreted based not only on theory but also on experience and practical knowledge, identifying possible limitations and opportunities for improvement that may not be evident through quantitative analysis alone. In addition, validation with experts ensures that the recommendations derived from the research are feasible and aligned with the needs and realities of the field of study [22]. The research was presented to 5 experts in the Healthcare domain, who discussed the results and evaluated their relevance and applicability. Individual sessions lasting approximately 45 minutes were held with each expert. During each session, slides were presented describing the study’s objective and goals and summarizing the results obtained. Ultimately, each expert answered four questions for each period analyzed (pre- and post-vaccination periods). The questions were:

1. Were the results presented surprising/unexpected?
2. What factors do you believe led to these results?
3. To what extent do these results contribute to formulating public policies in Latin America?
4. If you were a Public Health manager, how would these results contribute to creating guidelines for future respiratory diseases?

The objective was to ask questions that could be answered using a Likert scale, thus proving a quantifiable analysis and open-ended questions for qualitative analysis.

## Results and discussion

### Exploratory analysis

**Fig 1:**
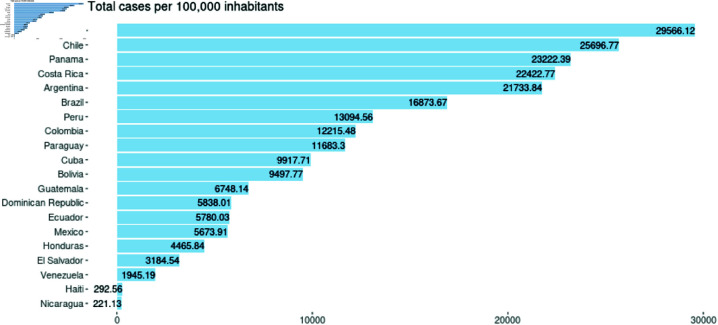
Total deaths per 100,000 pop. by country until October 15, 2022.

As seen in Fig 1, Peru had the most significant death rate per 100,000 pop. Until October 15, 2022, followed by Brazil and Chile. The number of deaths in Peru differs significantly from other countries. The countries with the fewest deaths are Nicaragua, Haiti, and Venezuela, in that order.

**Fig 2:**
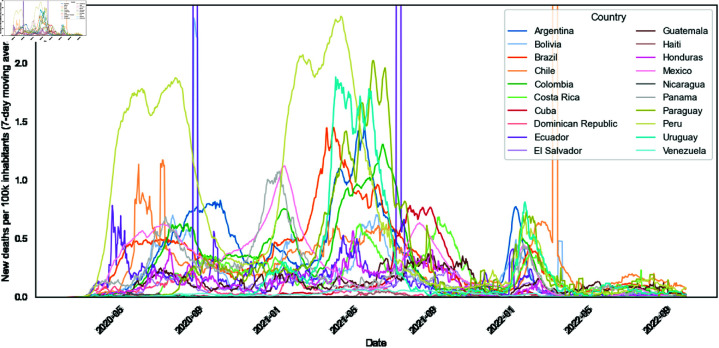
New deaths per 100k pop. (7-day moving average) time-series per LAC country. Chile, Ecuador, and El Salvador have had unexpected jumps in deaths from COVID-19, and on specific days in the Our World in Data database.

In Fig 2, it is possible to observe that each country had different waves of daily deaths and dimensions for this number of deaths.

**Fig 3:**
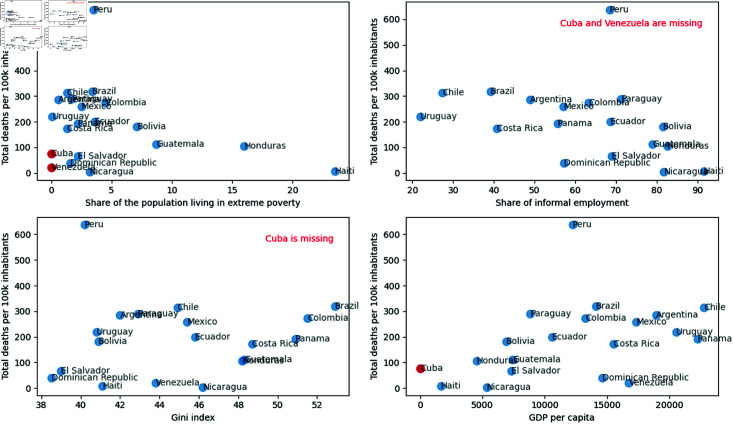
Relationship among total deaths per 100,000 pop. per country and the share of the population living in extreme poverty, the share of informal employment, the Gini index, and GDP per capita.

Haiti, with the largest share of the population living in extreme poverty and the largest share of the population in informal work, is shown in Fig 3. Brazil is the country with the most significant inequality, according to the Gini Index. Meanwhile, Haiti has the lowest GDP per capita. Among the ten socioeconomic and demographic variables, only Cuba and Venezuela have missing values or values equal to zero for some variables. The variables are:

- Cuba: share of informal employment, share of the population living in extreme poverty, Gini index, GDP per capita
- Venezuela: share of informal employment, share of the population living in extreme poverty

Furthermore, Cuba does not have Google data on population mobility, which is crucial for the research objective. Considering these points, Cuba and Venezuela were removed from the analysis.

### Clustering based on socioeconomic and demographic variables

Clustering was carried out to consider the different socioeconomic and demographic characteristics. However, before clustering, variables with VIF greater than ten were removed. Therefore, the variables selected for clustering were the Gini Index, average household size (number of members), median age of the population, share of the population living in extreme poverty, percentage of the population aged between 20 and 79 years who have type 1 or type 2 diabetes, population density. The variables removed were HDI, the average number of years of education received by people aged 25 and over, GDP per capita, and share of informal employment.

**Fig 4:**
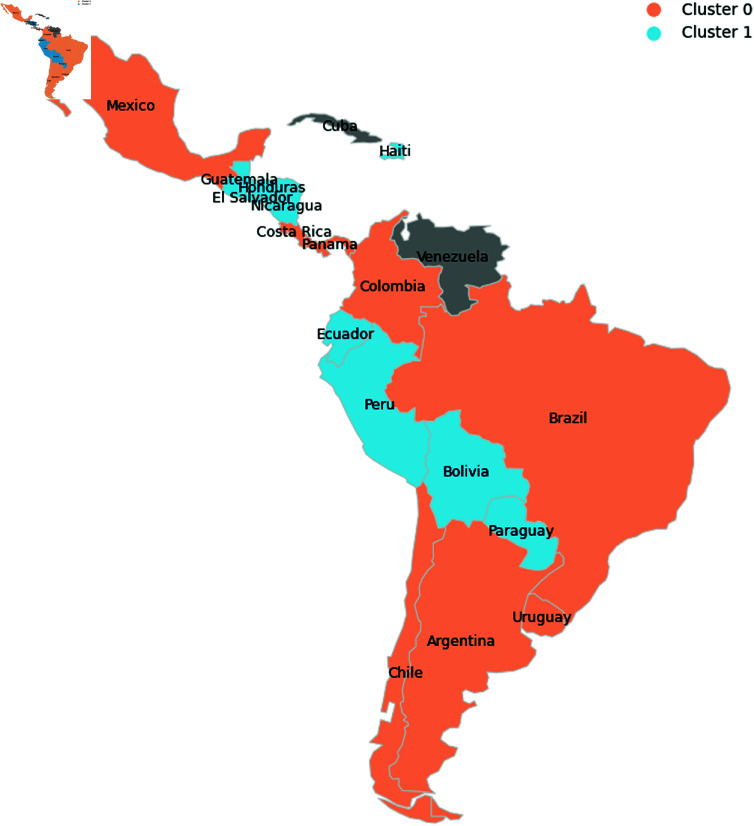
Map of LAC countries by cluster. Cluster 0 is a less vulnerable cluster compared to Cluster 1.

The DBSCAN and K-Means clustering methods were tested and evaluated based on the elbow method, silhouette index, and Calinski-Harabasz score. The principle of parsimony was also considered, prioritizing a simpler and more interpretable solution. Based on these evaluations, the chosen method was K-Means with 2 clusters, allowing for clear and robust comparative analysis between the groups. As shown in Fig 4, where cluster 0 comprises Argentina, Brazil, Chile, Colombia, Costa Rica, Mexico, Panama, and Uruguay. In contrast, cluster 1 comprises Bolivia, Dominican Republic, Ecuador, El Salvador, Guatemala, Haiti, Honduras, Nicaragua, Paraguay, and Peru.

**Fig 5:**
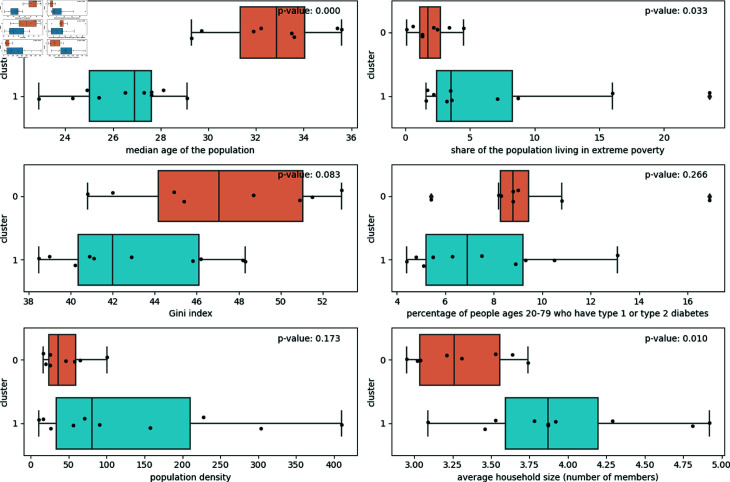
Boxplot of socioeconomic and demographic characteristics by cluster. Cluster 0 is a less vulnerable cluster compared to Cluster 1.

In Fig 5, it is possible to observe the distribution of clustering variables by cluster. The Mann-Whitney test indicated that, for a p-value of 0.05, the Gini index variables, the share of the population with diabetes, and population density do not have different medians. Furthermore, it is possible to observe that cluster 1 has a younger population than cluster 0, in addition to being the cluster with the largest population in extreme poverty and the largest average household size. In this way, cluster 0 is less vulnerable compared to cluster 1. Therefore, from now on, the present work will refer to cluster 0 as the less vulnerable countries and to cluster 1 as the more vulnerable ones.

An association analysis using the LMIC (Low- and Middle-Income Countries) classification from the World Bank revealed that the less vulnerable cluster contains more high-income countries. In contrast, the more vulnerable cluster includes lower-middle-income countries. This further corroborates the naming of the clusters as less and more vulnerable, reinforcing the socio-economic distinctions between them.

### Application of the lag between deaths and explanatory variables by cluster

It is important to lag deaths from interventions to assess the effectiveness of interventions during the pandemic [18, 23]. Therefore, a lag was applied between deaths and explanatory variables, which are interventions per cluster, where 21 to 49 days lags were tested for each cluster. The lags chosen for each cluster were:

- Less vulnerable cluster: 49 days
- More vulnerable cluster: 21 days

A possible interpretation for these lags is that more vulnerable cluster interventions had faster results than less vulnerable ones.

### Modeling deaths by cluster

The next step was to select the variables that would be used to explain deaths from COVID-19. For this, again, variables with VIF greater than ten were removed. In the end, the variables selected for modelling were school closures, restrictions on the size of meetings between people, closure of public transport, requirements to stay at home, restrictions on internal movement, debt/contract relief for families, income support for people who have lost their job or who cannot work, variation in mobility in supermarkets and pharmacies, variation in mobility in parks, variation in mobility in public transport, variation in mobility in places of work, variation in mobility in residential locations, percentage of the population vaccinated with at least two doses. Furthermore, the percentage of predominance of each variant per country was also included in the model to remove confounding effects associated with COVID-19 variants from the analysis.

In total, four models were constructed, and the SHAP Values technique was applied to each one of them:

1. Pre-vaccination period for the less vulnerable countries
2. Pre-vaccination period for the less vulnerable countries
3. Post-vaccination period for the more vulnerable countries
4. Post-vaccination period for the more vulnerable countries

### Analysis of the contribution of each feature per cluster

#### Pre-vaccination period.

**Fig 6:**
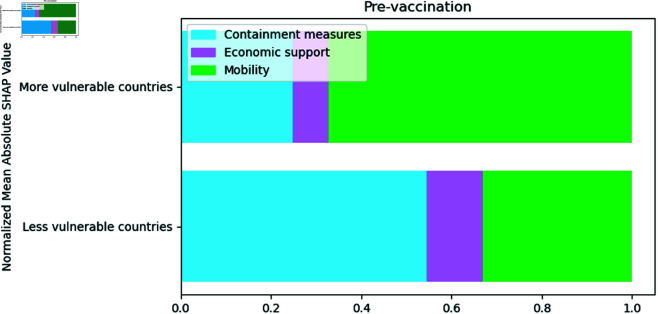
Normalized SHAP Values by cluster representing the relative importance of different features in more and less vulnerable countries. The larger the bar size, the greater the association with COVID-19 mortality

SHAP Values show that, when comparing less vulnerable countries with more vulnerable countries in the pre-vaccination period, it is possible to see from Fig 6 that the most important variables in less vulnerable countries were containment measures, while in more vulnerable countries, they were mobility variables. Furthermore, economic support variables were more important in less vulnerable than in more vulnerable countries.

**Fig 7:**
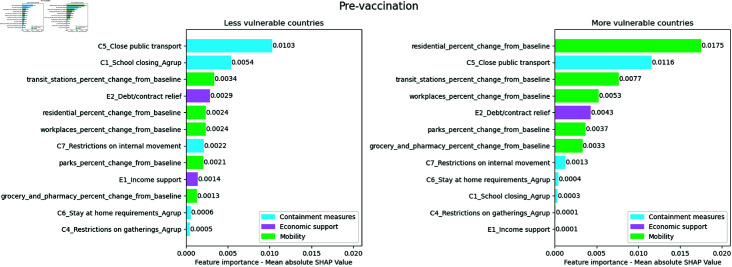
Absolute mean SHAP value per cluster for the pre-vaccination period. The numerical values next to each bar indicate the importance of the feature. Higher values reflect a greater association with COVID-19 mortality

In Fig 6 it was possible to observe that containment measures were the most influential in the less vulnerable countries, however through Fig 7 it is possible to observe that their relevance was driven mainly by the closure of public transport and schools, C5_Close public transport and C1_School closing_Agrup respectively. The most important variables for the most vulnerable countries were the variation in mobility in residential areas and the closure of public transport, residential_percent_change_from_baseline and C5_Close public transport. The closure of public transport was relevant in both clusters.

**Fig 8:**
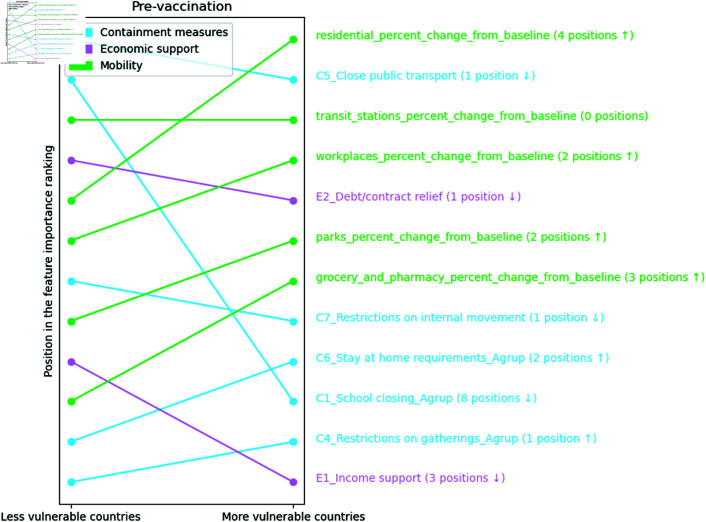
Ranking position of feature importance for each cluster in the pre-vaccination period. The numbers next to each feature indicate the change in its importance ranking, with arrows showing the direction of this change (up arrows indicating increase and down arrows indicating decrease)

Fig 8 shows that school closures, C1_School closing_Agrup, are the second most important variable in less vulnerable countries, but it drops eight positions in the importance ranking of more vulnerable countries. The mobility variation variable in transport stations, transit_stations_percent_change_from_baseline, has the same position in the importance ranking of both clusters. Both economic support variables are less important in more vulnerable countries than in less vulnerable ones.

**Fig 9:**
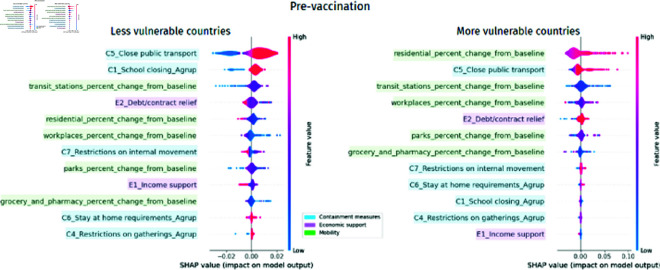
SHAP Values per cluster in the pre-vaccination period show each feature’s importance on COVID-19 mortality. X-Axis (SHAP Value) represents the contribution of each feature to the predicted COVID-19 mortality rate, where positive values indicate an increase in predicted mortality and negative values indicate a decrease in predicted mortality. Y-Axis lists the input features ranked by importance (average absolute SHAP value), with the most impactful features at the top. Color Gradient (blue to pink) represents the feature value for each country or observation, where pink indicates high feature values and blue indicates low feature values. Each dot corresponds to a specific country, showing how the value of a feature for that country influenced the model’s prediction of mortality

In Fig 9, it is possible to observe that in the pre-vaccination period, debt relief and economic support, E2_Debt/contract relief and E1_Income support respectively, are associated with reducing mortality from COVID-19 in less vulnerable countries. Some counterintuitive results indicate that the closure of public transport, the closure of schools, the increase in mobility in public transport stations, the increase in mobility in work areas, parks, stores, and pharmacies, and the restrictions on the size of meetings, C5_Close public transport, C1_School closing_Agrup, transit_stations_percent_change_from_baseline, workplaces_percent_change_from_baseline, parks_percent_change_from_baseline, grocery_and_pharmacy_percent_change_from_baseline and C4_Restrictions on gatherings_Agrup respectively, between people are associated with increased mortality.

For more vulnerable countries, unlike less vulnerable countries, the debt relief variable, E2_Debt/contract relief, is associated with increased mortality. The variation in mobility in residential areas, residential_percent_change_from_baseline, is the most important feature of the model, but it is always associated with an increase in mortality. The variation in mobility at transport stations, transit_stations_percent_change_from_baseline, is mainly associated with a reduction in mortality, as is the variation in mobility in parks, markets, and pharmacies, parks_percent_change_from_baseline and grocery_and_pharmacy_percent_change_from_baseline respectively.

#### Post-vaccination period.

**Fig 10:**
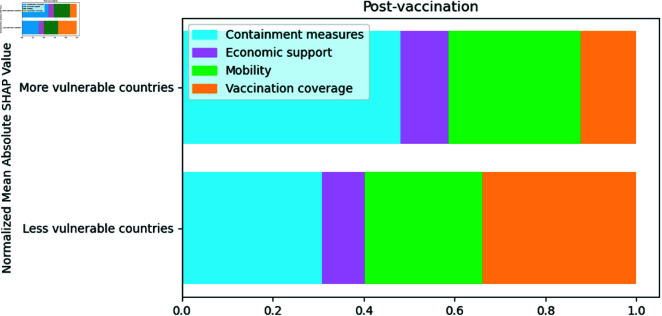
Normalized SHAP Values by cluster representing the relative importance of different features in more and less vulnerable countries. The larger the bar size, the greater the association with COVID-19 mortality

Fig 10 shows that the vaccination coverage variable in the post-vaccination period was the most important in less vulnerable countries. However, the most important variables for more vulnerable countries were the containment measure variables, followed by the mobility variables.

**Fig 11:**
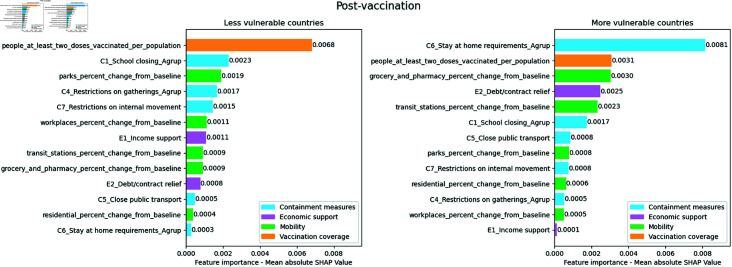
Absolute mean SHAP value per cluster for the post-vaccination period. The numerical values next to each bar indicate the importance of the feature. Higher values reflect a greater association with COVID-19 mortality

As seen in Fig 11, the vaccination variable, people_at_least_two_doses_vaccinated_per_population, in the least vulnerable countries is much more relevant than the others. Although in the most vulnerable countries, the most important type of variable is containment measures, the variable that drives this importance is the requirement to stay at home, C6_Stay at home requirements_Agrup. In the most vulnerable countries, the variables of stay at home requirements are more important than the vaccination coverage variable. In the least vulnerable countries, the most important containment measure was C1_School closing_Agrup.

**Fig 12:**
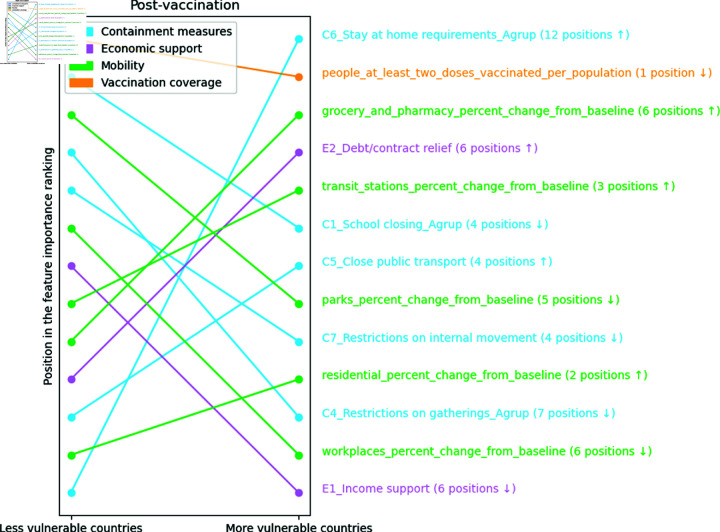
Ranking position of feature importance for each cluster in the post-vaccination period. The numbers next to each feature indicate the change in its importance ranking, with arrows showing the direction of this change (up arrows indicating increase and down arrows indicating decrease)

The economic support variable, E2_Debt/contract relief, drops six positions in the importance ranking of more vulnerable countries. The stay-at-home requirement variable, C6_Stay at home requirements_Agrup, is the most important for the more vulnerable and the least for the less vulnerable countries. Furthermore, the vaccination variable, people_at_least_two_doses_vaccinated_per_population, drops one position in the importance ranking of more vulnerable countries, as seen in Fig 12.

**Fig 13:**
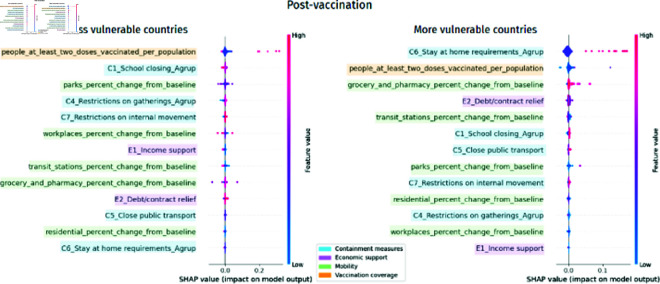
SHAP Values per cluster in the post-vaccination period show each feature’s importance on COVID-19 mortality. X-Axis (SHAP Value) represents the contribution of each feature to the predicted COVID-19 mortality rate, where positive values indicate an increase in predicted mortality and negative values indicate a decrease in predicted mortality. Y-Axis lists the input features ranked by importance (average absolute SHAP value), with the most impactful features at the top. Color Gradient (blue to pink) represents the feature value for each country or observation, where pink indicates high feature values and blue indicates low feature values. Each dot corresponds to a specific country, showing how the value of a feature for that country influenced the model’s prediction of mortality

In Fig 13, it is possible to observe that in the post-vaccination period, the percentage of the population vaccinated with at least two doses, people_at_least_two_doses_vaccinated_per_population, is associated with a reduction in mortality from COVID-19 in both less and more vulnerable countries. However, for more vulnerable countries, it is possible to observe that the requirements to stay at home, C6_Stay at home requirements_Agrup, are more relevant than vaccination and are associated with increased mortality.

Applying the SHAP Values technique to the four models revealed significant insights into the factors influencing COVID-19 mortality in different cluster contexts. It was possible to observe a marked distinction between less and more vulnerable countries in the pre-vaccination period. While containment measures emerged as the most important variables in less vulnerable countries, more vulnerable countries were mainly characterized by mobility variables. Although containment measures proved important in less vulnerable countries, their importance was driven mainly by the closure of public transport and schools.

However, when analyzing the post-vaccination period, it was possible to observe a significant change in dynamics. Vaccination coverage emerged as the most important variable in less vulnerable countries, reflecting the tangible benefits of vaccination in reducing mortality. Conversely, more vulnerable countries continued to be influenced mainly by containment measures and mobility variables. Some hypotheses for this are that less vulnerable countries have greater access to vaccines or greater vaccination coverage than more vulnerable countries.

Some findings were unexpected; for example, during the pre-vaccination phase in both less and more vulnerable countries, there was a correlation between increased deaths and public transport closures. A limitation of the current model is that it does not account for causality between variables. However, the lag was used to model the potential time course of the intervention effect.

Some hypotheses for these counterintuitive results are: the closure of public transport, for instance, may have hindered access to essential services, such as hospitals and healthcare clinics, especially for more vulnerable populations. Additionally, alternative transportation methods, such as carpooling or shared vehicles, may have increased exposure to the virus, contributing to higher mortality rates.

In the case of school closures, redistributing children’s and adolescents’ time to other environments, such as more intense domestic interactions or informal gatherings in uncontrolled settings, may have increased the risk of transmission within households or communities lacking adequate support to implement protective measures. Similarly, increased mobility in public areas, such as transport stations, parks, markets, and pharmacies, can be explained by a relaxation in adopting protective measures, such as mask use and social distancing. It is also possible that these areas attract economically active populations or individuals dependent on in-person work, who are more exposed to the virus and often have limited access to healthcare.

Furthermore, the association between economic support and lower mortality in less vulnerable countries can be explained by structural and institutional factors. In these countries, more robust institutions allow for the efficient implementation of economic programs, reaching the population in a timely and comprehensive manner. More resilient healthcare systems and higher formal employment rates facilitate more significant benefits for the population, amplifying the positive impact of economic support.

On the other hand, in more vulnerable countries, the effectiveness of economic support is limited by more profound structural inequalities, high levels of informality in the labor market, and insufficient resources. Program implementation is often delayed or has restricted coverage, and the benefits provided may not be sufficient to meet the basic needs of large segments of the population. These factors help explain the lack of an association between economic support and reduced mortality in these countries, emphasizing the need for policies adapted to the local context.

Despite some counterintuitive results, the model presented two that made sense given the history of COVID-19. It is known that the greater the percentage of the population with at least two doses of vaccine, the lower the number of deaths. There is also a hypothesis that when the government helps the population financially, people tend to stay home more, reducing the number of deaths.

### Assessment of the relevance and applicability of results with experts

Public health experts were invited to participate in panel discussions to assess the results of this research. They were selected based on their recognized experience and contribution to the medical field, possessing extensive understanding and knowledge on the subject under analysis. The experts of these panels revealed several valuable insights into the complexity and challenges faced in Public Health policy-making. One of the most discussed points was the relationship between correlation and causation. Experts highlighted the difficulty of establishing robust causal relationships from correlations observed in aggregated data. Three experts emphasized that the accuracy and validity of measures used to capture phenomena such as mobility and adherence to containment policies are crucial to avoid biases that could distort the results. [24] addresses the complexity and diversity of innovative lockdown measures during the COVID-19 pandemic. The authors highlight that while several localities have lockdown measures in place, they vary significantly in terms of definition and application, making it difficult to compare and assess their effectiveness across different regions and countries.

Another recurring theme in the discussions was the heterogeneity among regions and how this influences the effectiveness of Public Health policies. Four experts agreed that socioeconomic and demographic diversity across regions makes comparing policies and measures a significant challenge. One of the experts highlighted that “if there are already huge differences within Brazil, imagine comparing several countries”, highlighting the complexity of generalizing the results of Public Health policies. In addition, it was mentioned that adherence to containment measures varied significantly among regions, which may explain the unexpected results observed in some studies, as shown in [24]. The need to consider specific regional characteristics when formulating Public Health policies was widely recognized.

Vaccination was another central topic in the discussions. All experts agreed that vaccination was important in reducing mortality from COVID-19, especially in less vulnerable countries. However, it was also highlighted that the availability of and access to vaccines varied significantly among regions, influencing the effectiveness of vaccination campaigns. Three experts highlighted reducing inequity in access to the vaccine as a priority for future vaccination campaigns.

Economic support during the pandemic was also widely discussed. Two experts recognized that economic support measures, such as debt relief and income distribution, were essential to mitigate the economic impacts of the pandemic, especially in less vulnerable countries. However, they observed that the impact of these measures varied among regions. One expert mentioned that "the more economic support I provide, the lower my mortality is in these less vulnerable countries", highlighting the importance of adapting economic support policies to the specific needs of each region.

All experts indicated that the research results added value to the current literature since they shed light on issues that are not intuitive and that do not simply reproduce the results already known. Given the novelty of the results, the experts do not feel comfortable stating that they would adopt these results individually to define new public policies. However, it is worth further deepening the research to formulate public policies. One expert said, "I do not think it would be possible to use this result, for example, to formulate a policy. I think it brought a new perspective to the problem; all the points you raised here are points of conflict in the literature".

In summary, all experts pointed out that the most interesting result towards defining a Public Health policy was that vaccination coverage was associated with a reduction in COVID-19 mortality in both groups of countries and that economic support was associated with a decrease in COVID-19 mortality only in the less vulnerable countries, which implies that perhaps these countries were the ones that had the financial means to offer greater support. The expert discussions revealed the complexity and challenges in formulating public health policies based on the results of the COVID-19 pandemic analysis. The need for careful and contextualized analysis was widely recognized, considering regional heterogeneity and the relationship between correlation and causality. In addition, the importance of vaccination as an essential tool to mitigate the impacts of the pandemic was highlighted.

Feedback from public health experts was incorporated into the review of the research. These experts highlighted the need to make the distinction between correlation and causation explicit, particularly about the effectiveness of public policy interventions during the pandemic. They also highlighted the importance of considering regional heterogeneity and socioeconomic implications in their analyses, which led to a more critical review of the recommendations. This interaction with experts reinforced the robustness of the results and broadened the discussion on their practical implications in diverse socioeconomic settings.

## Conclusion

The first objective of the research was to understand which LAC countries are similar and how they are similar according to their socioeconomic and demographic characteristics. Two distinct groups of countries were detected, one characterized by a greater vulnerability than the other.

For the second objective of the research, the best lag between interventions (vaccination coverage, containment measures, economic support, and population mobility) and mortality from COVID-19 was determined as a time interval of 49 days for the less vulnerable countries, and of 21 days for the more vulnerable ones.

For the third research objective, we identified the most important interventions for understanding COVID-19 mortality and how they are associated with COVID-19 mortality in two distinct periods of the pandemic: pre-vaccination and post-vaccination. In the pre-vaccination period, containment measures were the most important interventions for mortality in the less vulnerable countries. In contrast, the variation in population mobility was highlighted for the more vulnerable countries. In the post-vaccination period, the most important interventions for mortality in the less vulnerable countries were vaccination coverage, while containment measures impacted the more vulnerable countries more.

Finally, the fourth objective, which aimed to provide insights into the definition of new Public Health guidelines for future respiratory diseases, was not fully achieved. The study did not succeed in proposing new public policies; however, health experts stated that, despite the results being counterintuitive, they bring new insights that spark relevant discussions and enrich the scientific debate on the topic. The main findings include two significant results: (1) vaccination coverage was consistently associated with a reduction in COVID-19 mortality in both groups of countries, highlighting vaccination as a critical factor in saving lives regardless of the level of vulnerability, and (2) economic support was associated with a reduction in COVID-19 mortality only in less vulnerable countries, suggesting that these countries had the financial capacity to implement more effective support measures. These findings underscore the importance of specific structural conditions for the effectiveness of Public Health interventions. In light of the unexpected and counterintuitive results, future work will apply XAI (Explainable AI) techniques and causal inference analyses to deepen the understanding of the dynamics observed in the study and enable further advances in the field.

**Fig 14:**
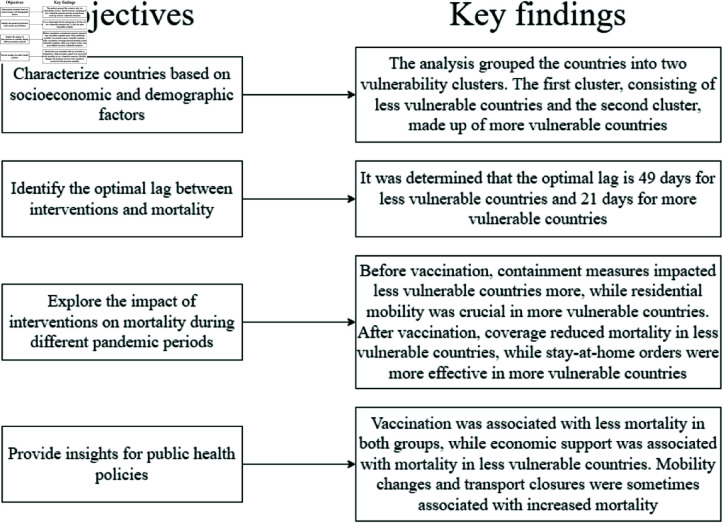
Summary of study objectives and key findings.

Fig 14 summarizes the objectives and key findings of the study presented in this conclusion, providing a visual overview of the analyses conducted and the results obtained.

Although the research identified significant associations between the variables, it is essential to clarify that this study exclusively addresses correlation, not causation. The observed relationships may be influenced by uncontrolled confounders or omitted variables that simultaneously affect the variables of interest. Causal inference techniques were not applied in this study; however, future analyses could use such methodologies to further and more accurately explore the results obtained, helping to distinguish between observed associations and potential causal relationships. Additionally, it is important to highlight the difficulty of isolating the effects of different interventions due to the simultaneity of the measures analyzed.

One point of discussion in the research is the heterogeneity of the regions studied and its impact on the generalization of the results. Latin America is characterized by substantial socioeconomic, cultural, and geographic diversity, which may influence the observed effects of the variables analyzed differently. This heterogeneity may limit the generalization of the results obtained to the entire region since conclusions derived from a specific context may not directly apply to other contexts with different characteristics. For example, Public Health policies that have proven effective in a country with robust infrastructure may not have the same impact in countries with underfunded Healthcare systems.

An important limitation of this study is the potential influence of confounding variables, such as healthcare infrastructure, testing rates, and potential reporting biases in the analyzed countries. These variables may impact both the relationship between the interventions analyzed and mortality and the interpretation of the results. For instance, countries with more robust healthcare systems may exhibit lower mortality rates regardless of implemented interventions. In contrast, limited testing rates might underestimate the actual number of cases and deaths, distorting observed associations. We acknowledge that the absence of direct adjustments for these variables may introduce biases into the results, and we suggest that future studies incorporate these considerations to deepen the understanding of the analyzed relationships.

## References

[1] Kwok KO, Tang A, Wei VWI, Park WH, Yeoh EK, Riley S. Epidemic models of contact tracing: systematic review of transmission studies of severe acute respiratory syndrome and Middle East respiratory syndrome. Comput Struct Biotechnol J. 2019;17:186–94. doi: 10.1016/j.csbj.2019.01.003 30809323 PMC6376160

[2] Fauci AS, Lane HC, Redfield RR. Covid-19 - navigating the uncharted. N Engl J Med. 2020;382(13):1268–9. doi: 10.1056/NEJMe2002387 32109011 PMC7121221

[3] Burki T. COVID-19 in Latin America. Lancet Infect Dis. 2020;20(5):547–8. doi: 10.1016/S1473-3099(20)30303-0 32311323 PMC7164892

[4] Garcia PJ, Alarcón A, Bayer A, Buss P, Guerra G, Ribeiro H, et al. COVID-19 response in Latin America. Am J Trop Med Hyg. 2020;103(5):1765–72. doi: 10.4269/ajtmh.20-0765 32940204 PMC7646820

[5] Pablos-Méndez A, Vega J, Aranguren FP, Tabish H, Raviglione MC. Covid-19 in Latin America. BMJ. 2020;370:m2939. doi: 10.1136/bmj.m2939 32718938

[6] Litewka SG, Heitman E. Latin American healthcare systems in times of pandemic. Dev World Bioeth. 2020;20(2):69–73. doi: 10.1111/dewb.12262 32282974 PMC7262025

[7] Paniz-Mondolfi AE, Sordillo EM, Márquez-Colmenarez MC, Delgado-Noguera LA, Rodriguez-Morales AJ. The arrival of SARS-CoV-2 in Venezuela. Lancet. 2020;395(10236):e85–6. doi: 10.1016/S0140-6736(20)31053-9 32380043 PMC7198211

[8] Peres IT, Bastos LSL, Gelli JGM, Marchesi JF, Dantas LF, Antunes BBP, et al. Sociodemographic factors associated with COVID-19 in-hospital mortality in Brazil. Public Health. 2021;192:15–20. doi: 10.1016/j.puhe.2021.01.005 33607516 PMC7836512

[9] Bargain O, Aminjonov U. Poverty and COVID-19 in Africa and Latin America. World Dev. 2021;142:105422. doi: 10.1016/j.worlddev.2021.105422 33612919 PMC7885669

[10] Bennett M. All things equal? Heterogeneity in policy effectiveness against COVID-19 spread in chile. World Dev. 2021;137:105208. doi: 10.1016/j.worlddev.2020.105208 32994662 PMC7513907

[11] Martinez-Valle A. Public health matters: why is Latin America struggling in addressing the pandemic?. J Public Health Policy. 2021;42(1):27–40. doi: 10.1057/s41271-020-00269-4 33510400 PMC7841039

[12] Chan IL, Mowson R, Alonso JP, Roberti J, Contreras M, Velandia-González M. Promoting immunization equity in Latin America and the Caribbean: case studies, lessons learned, and their implication for COVID-19 vaccine equity. Vaccine. 2022;40(13):1977–86. doi: 10.1016/j.vaccine.2022.02.051 35221122 PMC8841228

[13] Bastos LSL, Aguilar S, Rache B, Maçaira P, Baião F, Cerbino-Neto J, et al. Primary healthcare protects vulnerable populations from inequity in COVID-19 vaccination: an ecological analysis of nationwide data from Brazil. Lancet Reg Health Am. 2022;14:100335. doi: 10.1016/j.lana.2022.100335 35991675 PMC9381845

[14] Tavares FF, Betti G. The pandemic of poverty, vulnerability, and COVID-19: evidence from a fuzzy multidimensional analysis of deprivations in Brazil. World Develop. 2021;139:105307. doi: 10.1016/j.worlddev.2020.105307

[15] O’brien RM. A caution regarding rules of thumb for variance inflation factors. Qual Quant. 2007;41(5):673–90. doi: 10.1007/s11135-006-9018-6

[16] Ikotun AM, Ezugwu AE, Abualigah L, Abuhaija B, Heming J. K-means clustering algorithms: a comprehensive review, variants analysis, and advances in the era of big data. Inf Sci. 2023;622:178–210. doi: 10.1016/j.ins.2022.11.139

[17] Ester M, Kriegel H, Sander J, Xu X. A density-based algorithm for discovering clusters in large spatial databases with noise. In: Proceedings of the Second International Conference on Knowledge Discovery and Data Mining. AAAI Press. 1996. p. 226–31.

[18] Costa D, Rohleder S, Bozorgmehr med K. Impact of non-pharmaceutical interventions on COVID-19 incidence and deaths: cross-national natural experiment in 32 European countries. Cold Spring Harbor Laboratory. 2022. doi: 10.1101/2022.07.11.22277491 PMC11361163 39198794

[19] Coxe S, West SG, Aiken LS. The analysis of count data: a gentle introduction to poisson regression and its alternatives. J Pers Assess. 2009;91(2):121–36. doi: 10.1080/00223890802634175 19205933

[20] Chen T, Guestrin C. XGBoost. In: Proceedings of the 22nd ACM SIGKDD International Conference on Knowledge Discovery and Data Mining. ACM. 2016. p. 785–94. doi: 10.1145/2939672.2939785

[21] Lundberg S, Lee S. A unified approach to interpreting model predictions. CoRR. 2017. doi: 10.5555/3295222.3295230

[22] Lu Z, Wang Y. Teaching CORnet human fMRI representations for enhanced model-brain alignment. Cogn Neurodyn. 2025;19(1):61. doi: 10.1007/s11571-025-10252-y 40242427 PMC11999921

[23] Li H, Wang L, Zhang M, Lu Y, Wang W. Effects of vaccination and non-pharmaceutical interventions and their lag times on the COVID-19 pandemic: comparison of eight countries. PLoS Negl Trop Dis. 2022;16(1):e0010101. doi: 10.1371/journal.pntd.0010101 35025865 PMC8757886

[24] Fabio I, Amaral G, Griffo C, Baião F, Guizzardi G. What exactly is a lockdown?: Towards an Ontology-based modeling of lockdown interventions during the COVID-19 pandemic. In: ONTOBRAS 2021: Ontology Research in Brazil. CEUR; 2021. p. 151–64.

